# Comparing efficacy of reduced-fluence and standard-fluence photodynamic therapy in the treatment of polypoidal choroidal vasculopathy

**DOI:** 10.1186/s12886-020-01419-8

**Published:** 2020-04-15

**Authors:** Wei Kiong Ngo, Wai Kitt Chee, Colin S. Tan, Tock Han Lim

**Affiliations:** 1 National Healthcare Group Eye Institute, Tan Tock Seng Hospital, 11 Jalan Tan Tock Seng, Singapore, 308433 Singapore; 2 Fundus Image Reading Centre, National Healthcare Group Eye Institute, Singapore, Singapore

**Keywords:** Comparing reduced and standard-fluence photodynamic therapy

## Abstract

**Background:**

The EVEREST II study reported superior polyp closure rates and visual outcomes using combination standard photodynamic therapy (PDT) with intravitreal ranibizumab in the treatment of polypoidal choroidal vasculopathy (PCV). The optimal PDT protocol remains controversial and it is postulated that less intensive PDT strategies may reduce complications. We aimed to compare the efficacy of reduced and standard-fluence PDT.

**Methods:**

Case-control review of 38 consecutive PDT-naïve macular PCV patients who underwent verteporfin PDT using one of two PDT regimens at a tertiary referral centre in an Asian population. Comparison of outcomes between standard-fluence PDT (light dose, 50 J/cm2; dose rate, 600 mW/cm2; wavelength, 689 nm PDT applied to the treatment eye for 83 s) and reduced-fluence PDT (light dose, 25 J/cm2; dose rate, 600 mW/cm2; wavelength, 689 nm PDT applied to the treatment eye for 42 s). Primary outcome measure was best corrected LogMAR visual acuity (VA). Secondary outcome measures included OCT measurements such as central retinal thickness (CRT), height of subfoveal sub-retinal fluid (SRF), central choroid thickness (CCT), mean number of PDT treatments needed, mean number of anti-VEGF injections needed, polyp closure and recurrence rates.

**Results:**

Of these 38 eyes of 38 patients, an equal number of eyes (19 in each arm) were treated with standard-fluence and reduced-fluence PDT. Mean letter gain at 12 months for the standard-fluence group was 6.0 compared to 4.3 letters for the reduced-fluence group (*p* = 0.61). Similar results were observed at all time points. There was no statistically significant difference between the retinal and choroidal anatomical OCT outcomes, rates of polyp closure and recurrences between the two PDT regimens.

**Conclusions:**

Reduced-fluence PDT was comparable to standard-fluence PDT in the treatment of PCV in terms of visual gains, clinical and anatomical OCT outcomes.

## Background

Polypoidal choroidal vasculopathy (PCV) is a variant of exudative age-related macular degeneration (AMD) and is characterized by abnormal vascular networks and terminal dilatations which form the polyps [1]. It is an important variant of AMD especially in Asians, with the prevalence described to be as high as 55% amongst those with wet AMD [2].

While typical AMD is treated with anti-VEGF monotherapy, the EVEREST study reported superior rates of complete polyp regression among eyes with PCV when verteporfin photodynamic therapy (PDT) was administered, either as monotherapy or in combination with intravitreal ranibizumab, compared to intravitreal ranibizumab alone [3]. These findings were confirmed in the subsequent EVEREST II study, [4] which also reported superior visual gains in the group treated with combination PDT and intravitreal ranibizumab. The total number of intravitreal ranibizumab injections needed in the 2-year follow up duration was also lower in the combination (PDT and intravitreal ranibizumab) arm compared to the monotherapy arm (intravitreal ranibizumab) [4]. In the EVEREST study, verteporfin was dosed at 6 mg/m^2^, with standard (light dose, 50 J/cm^2^; dose rate, 600 mW/cm^2^; wavelength, 689 nm) PDT applied to the treatment eye for 83 s [3, 5].

In the literature, standard PDT have been reported to result in sight threatening complications such as subretinal, vitreous and suprachoroidal haemorrhage, as well as tears and rips of the retinal pigment epithelium [6–9]. PDT is also believed to cause thrombosis of the choriocapillaris leading to choroidal ischemia which may lead to upregulation of vascular endothelial growth factors (VEGF) expression and ultimately results in chorioretinal thinning and atrophy [10–12].

To mitigate the risk of complications using standard PDT, less intensive protocols for PDT treatment have been suggested. They include reducing the dose of verteporfin administered or reducing one of the laser parameters (dose rate or duration of laser application) to achieve a reduction in the laser fluence (light dose) [13–20]. Various authors have described treatment success with these less intensive protocols which theoretically reduces the risk of complications [13–20]. Several studies have reported the success of reduced-fluence PDT in the treatment of PCV [13–16, 18–20]. However, all except one of these reports are single-arm interventional series involving only the reduced-fluence arm, without the inclusion of a standard-fluence arm for comparison [14–16, 18–20]. Only Fan et al. compared the outcomes between reduced-fluence and standard-fluence PDT [13].

In this study, we aimed to compare the clinical efficacy of reduced-fluence PDT to standard-fluence PDT in the treatment of PCV in a series of Asian patients.

## Methods

This study was approved by the National Healthcare Group Domain Specific Review Board (DSRB) and conformed to the Tenets of the Declaration of Helsinki. Written, informed consent was obtained from patients prior to initiation of treatment.

We retrospectively reviewed 38 eyes of 38 consecutive PDT-naïve patients diagnosed with macular PCV who underwent verteporfin PDT from January 2011 to December 2013 in a tertiary Ophthalmology referral centre in central Singapore. In our centre, we practise achieving reduced-fluence PDT by reducing the duration of laser application. PCV was diagnosed by reading-center certified retinal specialists using dynamic indocyanine green (ICGA) and fluorescein (FA) angiograms (Heidelberg Spectralis, Heidelberg Engineering GmbH, Heidelberg, Germany) using the diagnostic criteria adopted by the EVEREST and EVEREST II studies [3–5, 21–23]. Spectral-domain optical coherence tomography (OCT) (Heidelberg Spectralis, Heidelberg Engineering GmbH, Heidelberg, Germany) was performed in all cases to obtain thickness measurements used in the secondary outcomes described below.

All cases were reviewed by graders from the Fundus Image Reading Center, National Healthcare Group, to confirm the diagnosis of PCV, using standardized diagnostic criteria. PCV was diagnosed based on early subretinal ICGA hyperfluorescence, occurring within the first 6 min, and at least one of the following diagnostic criteria: 1) Nodular appearance of the polyp on stereoscopic viewing, 2) Hypofluorescent halo around the nodule, 3) Abnormal vascular channel(s) supplying the polyps, 4) Pulsatile filling of polyps, 5) Orange sub-retinal nodules corresponding to the hyperfluorescent area on ICGA, 6) Massive submacular haemorrhage [3–5, 21–23]. Cases were classified as macular PCV when more than half of the lesion is within a 6000 μm diameter zone centered over the fovea avascular zone [3–5, 22].

All cases were treated using PDT with or without intravitreal anti-vascular endothelial growth factors (VEGF) injections. For PDT (Carl Zeiss Visulas 690 s PDT system, Carl Zeiss Meditec AG, Oberkochen, Germany), the treatment zone(s) included all polyps and the associated branching vascular network, whilst avoiding the optic disc. Two PDT regimens were used in our centre, namely reduced-fluence PDT and standard-fluence PDT:

1. Standard-fluence PDT (light dose, 50 J/cm^2^): dose rate, 600 mW/cm^2^; wavelength, 689 nm PDT applied to the treatment eye for 83 s
2. Reduced-fluence PDT (light dose, 25 J/cm^2^): dose rate, 600 mW/cm^2^; wavelength, 689 nm PDT applied to the treatment eye for 42 s

All cases described in this study were treated by two experienced fellowship trained retinal specialists (CST and THL). The choice of PDT regimen (standard-fluence or reduced-fluence) and whether it was done in combination with anti-VEGF or as monotherapy initially was based on a multi-modal imaging approach where disease factors such as retinal thickness and height of subretinal fluid measured using OCT at presentation was taken into account, combined with the best clinical judgement of the managing retinal specialist. Additional anti-VEGF injections and PDT treatments were performed if there was persistence or recurrence of disease activity (defined as the presence of new hemorrhages, any fluid in the intra-retinal or sub-retinal compartments) based on multi-modal imaging and clinical judgement.

Eyes with concomitant diseases that may affect visual outcomes such as diabetic macular edema and glaucoma were excluded from this analysis.

Primary outcome measure in this study was best corrected LogMAR visual acuity (VA) while secondary outcome measures included OCT measurements such as central retinal thickness (CRT), height of subfoveal sub-retinal fluid (SRF), central choroid thickness (CCT), mean number of PDT treatments needed, mean number of anti-VEGF injections needed, polyp closure and recurrence rates. CRT in the central 1-mm ETDRS subfield was automatically generated in the viewing software after manual correction of segmentation errors. The height of SRF and CCT were manually measured sub-foveally using in-built calipers available in the viewing software (Heidelberg Eye Explorer version 1.10.0.0, Heidelberg Engineering GmbH, Heidelberg, Germany). The other secondary outcome measures were obtained and analysed from clinical case sheet data.

Statistical analysis was performed using the SPSS software version 16 (IBM SPSS, Armonk, New York, USA). Continuous variables were analysed using t-tests, while categorical variables using chi-square tests.

## Results

### Demographics

Thirty-eight eyes of 38 patients were included in this study. Of these, 23 (60.5%) were males while 15 (39.5%) were females. The mean age of the patients was 70.0 years (range 50–86 years; S.D. ± 7.9). These patients were followed-up for a mean duration of 24.0 months (range 6–48 months). Of these, 19 (50.0%) eyes were treated with standard-fluence PDT while 19 eyes (50.0%) were treated using reduced-fluence PDT.

Patients were divided into two groups for analysis based on their PDT treatment regime. We compared baseline age, visual acuity, and OCT characteristics of both groups and found no statistically significant differences in baseline characteristics between the two groups (Table 1).

**Table 1:** Comparison of baseline characteristics between PDT treatment regimes

Baseline characteristic	PDT treatment regime		***P*** value
	Standard-fluence PDT (***n*** = 19)	Reduced-fluence PDT (***n*** = 19)	
Age (years)	68.4 (SD ± 7.6)	71.5 (SD ± 8.2)	0.233
LogMAR visual acuity	0.41 (SD ± 0.23)	0.32 (SD ± 0.21)	0.221
Central retinal thickness (μm)	406.4 (SD ± 118.4)	342.4 (SD ± 100.6)	0.081
Height of subretinal fluid (μm)	90.5 (SD ± 121.8)	87.3 (SD ± 86.1)	0.926
Central choroidal thickness (μm)	234.6 (SD ± 82.0)	220.1 (SD ± 106.2)	0.640

### Visual outcome

**Fig. 1:**
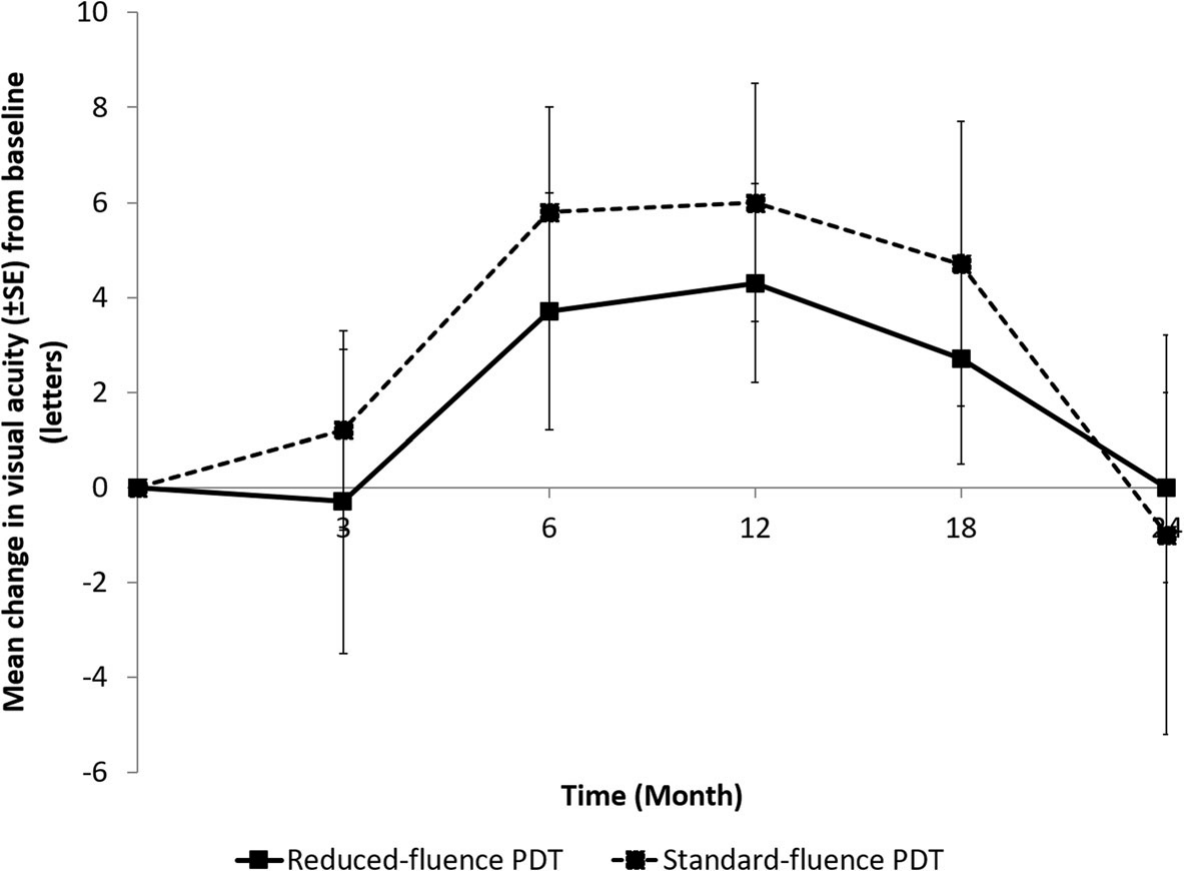
Mean change in visual acuity (±SE) from baseline (letters)

Overall, both groups gained vision compared to baseline during the duration of the study (Fig. 1). The mean letter gain in LogMAR VA was most notable between 6 to 18 months, but by the end of 24 months, the mean VA returned to baseline. The mean letter gain for the standard-fluence group was 1.2, 5.8, 6.0, 4.7 and − 1.0 (loss) letters compared to − 0.3 (loss), 3.7, 4.3, 2.7 and 0 (no change) letters for the reduced-fluence group at 3, 6, 12, 18 and 24 months respectively. There was a trend that standard-fluence group gained more vision than the reduced-fluence group, particularly between 3 to 18 months. However, there was no statistical significant difference in mean gain of VA between the two groups (*p* = 0.54–0.81) at all time points.

### OCT characteristics

**Fig. 2:**
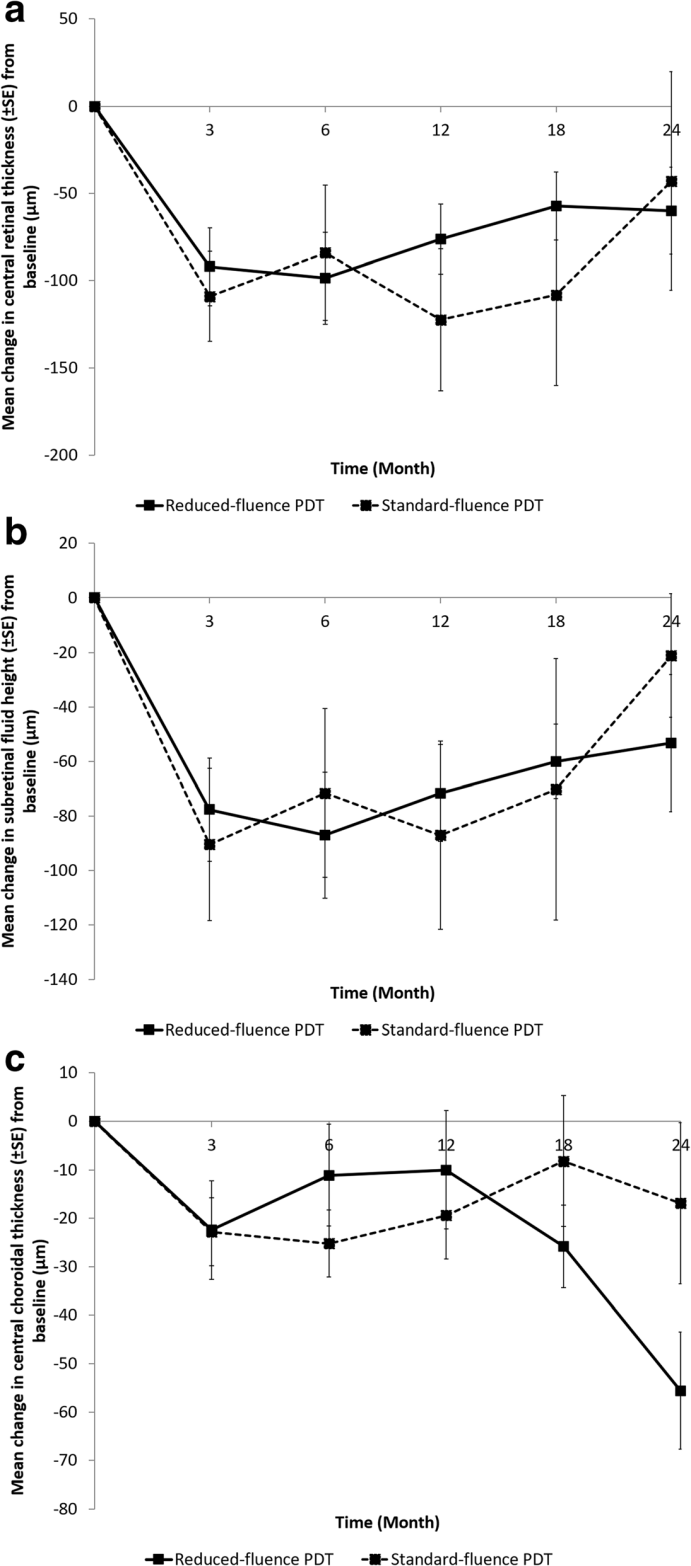
**a**: Mean change in central retinal thickness (±SE) from baseline (μm). **b**: Mean change in subretinal fluid height (±SE) from baseline (μm). **c**: Mean change in central choroidal thickness (±SE) from baseline (μm)

The mean CRT (Fig. 2a), SRF height (Fig. 2b) and CCT (Fig. 2c) at baseline and follow up visits up to 24 months are illustrated.

Reduction in mean CRT compared to baseline was comparable in both groups up to 24 months, with no significant differences between both groups. The mean decrease in CRT was 109.0 μm, 84.1 μm, 122.5 μm, 108.2 μm and 42.9 μm in the standard-fluence group compared to 92.0 μm, 98.6 μm, 76.3 μm, 57.3 μm and 60.0 μm in the reduced-fluence group at 3, 6, 12, 18 and 24 months respectively (*p* = 0.32–0.77).

Similarly, the decrease in height of SRF was similar between both groups at all time points. The decrease in SRF in the standard-fluence group was 90.5 μm, 71.6 μm, 87.1 μm, 70.3 μm and 21.2 μm compared to 77.7 μm, 87.1 μm, 71.6 μm, 59.9 μm and 53.3 μm in the reduced-fluence group at 3, 6, 12, 18 and 24 months respectively (*p* = 0.41–0.82).

The magnitude of change in CCT between both groups from their baseline measurements were similar at all time points. The decrease in CCT in the reduced-fluence group was 22.8 μm, 25.2 μm, 19.4 μm, 8.2 μm and 16.9 μm compared to 22.4 μm, 11.1 μm, 10.0 μm, 25.8 μm and 55.6 μm in the standard-fluence group at 3, 6, 12, 18 and 24 months respectively (*p* = 0.14–0.97).

### Polyp closure rates

In 25 patients (16 in the standard-fluence group and 9 in the reduced-fluence group), ICGA was repeated approximately 3 months after initial PDT to assess the activity of the PCV lesions. Polyp closure was 81.3% (13/16) in the standard-fluence group while it was 77.8% (7/9) in the reduced-fluence group. There was no statistical significant difference between both groups (*p* = 0.84).

### Number of anti-vascular endothelial growth factor injections, repeat photodynamic therapy and recurrences

Overall, 94.7% (18/19) of patients in the standard-fluence PDT group and 89.5% (17/19) of those in the reduced-fluence PDT group received at least one anti-vascular endothelial growth factor injection. Only 1 (5.3%) and 2 (10.5%) patients in the standard-fluence and reduced-fluence groups received PDT monotherapy respectively (*p* = 0.55). The average number of anti-vascular endothelial growth factor injections administered during the course of the follow up (2 years) was similar between the two groups. The standard-fluence group needed a mean of 5.0 injections while the reduced-fluence group required a mean of 6.0 injections (*p* = 0.50).

Comparing both groups, there was also no significant difference between the number of additional PDT treatments required (*p* = 0.40) within the 2 years. The mean (median) number of repeat PDT required was 0.37 (0) in the standard-fluence group compared to 0.16 (0) in the reduced-fluence group.

The rate of recurrence (over 2 years) of active PCV lesions after clinical quiescence was 42.1% (8/19) in the standard-fluence group and 36.8%% (7/19) in the reduced-fluence group, although this difference was not statistically significant (*p* = 0.74).

### Complications

None of the patients in either the standard-fluence PDT group nor the reduced-fluence PDT group suffered from any major complications such as massive hemorrhages, retinal pigment epithelial tears or severe visual loss secondary to choroidal ischemia after PDT in this study.

## Discussion

Standard PDT has been well described in the treatment of PCV with polyp closure rates exceeding 80% in some studies [6, 7]. The EVEREST and EVEREST II studies also reported superior rates of complete polyp regression using standard PDT in combination with intravitreal ranibizumab when compared to intravitreal ranibizumab monotherapy [3, 4]. However, massive hemorrhages, exudation and choroidal ischemia after standard PDT have been reported to result in vision loss in 11.0–30.8% of eyes [8–12]. In contrast, our study and the EVEREST studies reported no major complications such as massive hemorrhages or severe loss of vision secondary to choroidal ischemia occurring after PDT [3, 4]. To reduce the unwanted effects of PDT, there are increasing reports describing less intensive alternative PDT regimens to reduce either the laser fluence (light dose) or the dosage of verteporfin administered.

PDT works on the basis of the initiation of photochemistry at the target sites. Intravenous application of the photosensitizer (verteporfin) followed by laser application for absorption by the sensitizer activates a cascade of chemical reactions leading to cytotoxic free radical formation that result in endothelial damage, blood flow stasis and eventual shutdown of vasculature [24]. Preferential concentration of verteporfin in the abnormal vasculature forming the polyps and branching vascular network minimizes collateral damage [24]. There is a direct correlation to the phototoxic effects with the drug dose and light dose. For example, if the drug dose is lowered, a higher light dose (if dose rate is kept constant, a longer duration of light exposure is required) would be required to achieve similar effects and vice versa. Therefore, the phototoxic effect can be altered by either adjusting the light dose or the drug dose [24]. In our study, we chose to alter the fluence (light dose) by reducing the duration of laser application while keeping the dose rate constant. One practical advantage of reducing the duration of laser application, instead of the dose rate, to achieve reduced-fluence was that it reduces the total amount of treatment time required, especially in bilateral cases or in cases where more than one treatment area is required in the affected eye.

In our study, we found that reduced-fluence PDT was comparable to standard-fluence PDT in the treatment of PCV in terms of visual outcomes. Most notably between 3 to 18 months, there was a trend that the standard-fluence group gain more letters than the reduced-fluence group, although the difference was not statistically significant. Overall, both groups gained letters between 3 to 18 months, but by 24 months, the mean VA reverted to baseline. This can be attributed to the fact that approximately 40% of eyes in both groups had recurrence of disease activity within the 2 years follow up. Anatomically on OCT, reduced-fluence PDT also resulted in similar reductions in CRT, SRF height and CCT when compared to standard-fluence PDT.

The EVEREST and EVEREST II studies demonstrated that the value of standard PDT in the treatment of PCV lies in its superior efficacy to close the active polypoidal lesions over anti-VEGF monotherapy [3, 5]. Therefore, in our study, we analysed a subgroup of patients who had ICGA repeated at approximately 3 months after initial PDT to describe the polyp closure rates. We again found that reduced-fluence PDT was equally efficacious compared to standard-fluence PDT in achieving polyp closure, with rates of 77.8% in the reduced-fluence group, which is comparable to those reported in the clinical trials using standard PDT. In addition to improvements in VA, we believe that polyp regression is important due the risk of recurrent haemorrhages from patent polyps. In some cases, massive submacular haemorrhage may occur, with significant loss of VA [21, 25]. Reduced-fluence PDT was also comparable to standard-fluence PDT when we compared with respect to mean and median number of anti-VEGF and additional PDT. Recurrence rates between both groups were also similar with no statistically significant difference.

Only two other studies have described using reduced-fluence PDT by reducing duration of laser exposure to treat PCV [13, 15]. They reported improvements of LogMAR VA ranging from 0.17–0.24, [13, 15] mean central retinal thickness decrease of 208 μm, [15] and polyp regression rate of 58.3–79% [13, 15]. Some studies have described favourable outcomes with the use of reduced dose rate for PDT and reduced-dosage of verteporfin used in standard PDT. Wong et al. reported LogMAR VA improvement of 0.23 and polyp regression rate of 42.1% in the use of half-dose verteporfin PDT [17]. Other authors have reported the use of reduced dose rate for PDT resulting in LogMAR VA improvements ranging from 0.13–0.3, mean central foveal thickness decreases of 87–281 μm, and polyp regression rates between 71.4–92.8% [14, 16–20]. These results are comparable to those reported in our study. Fan also showed that reduced-fluence PDT combined with intravitreal bevacizumab had improved best corrected visual acuity at 6 months, with less lipid exudation and a smaller area of retinal haemorrhage when compared to standard-fluence PDT alone [13].

One of the main safety concerns of standard PDT is that it results in thrombosis of the choriocapillaris leading to choroidal ischemia, chorioretinal thinning and atrophy [11, 12]. Damage to choriocapillaries after standard PDT resulting in hypoperfusion areas shown on ICGA has been reported [12]. It would be ideal to compare size of ICGA-determined hypoperfusion areas on the fundus between the two treatment arms but this was not a primary aim of our study. Instead, we measured CCT as a surrogate marker for chorioretinal atrophy over a 24 months duration. We found no significant difference in the reduction of CCT between both treatment groups.

This study is not without limitations. Our study was a retrospective analysis and subject to the selection bias associated with such a study. The sample size was also small and there was no randomization in the process of assigning the treatment arms. To address these, we included equal numbers of eyes in both treatment arms with similar baseline characteristics to make a case-control comparison. Additionally, this is one of few studies which performed a head to head comparison of reduced-fluence PDT to standard-fluence PDT in the treatment of PCV and its results offer us some initial “real-world” insights into the efficacy of different PDT treatment regimens.

In conclusion, the results of our review showed that reduced-fluence PDT was at least comparable to standard-fluence PDT in the treatment of PCV. Future randomised controlled studies to determine the efficacy and long-term safety of reduced-fluence compared to standard-fluence PDT regimens are warranted.

## Data Availability

The datasets used and/or analysed during the current study are available from the corresponding author on resonable request.
